# Statistical adjustment of genotyping error in a case–control study of childhood leukaemia

**DOI:** 10.1186/1471-2288-12-141

**Published:** 2012-09-13

**Authors:** Matthew N Cooper, Nicholas H de Klerk, Kathryn R Greenop, Sarra E Jamieson, Denise Anderson, Frank M van Bockxmeer, Bruce K Armstrong, Elizabeth Milne

**Affiliations:** 1 Telethon Institute for Child Health Research, Centre for Child Health Research, University of Western Australia, P.O. Box 855, West Perth, 6872, WA, Australia; 2 School of Pathology and Laboratory Medicine, University of Western Australia, Path West Biochemistry, Royal Perth Hospital, Crawley, WA, Australia; 3 Sydney School of Public Health, University of Sydney, Camperdown, NSW, Australia

**Keywords:** Biostatistics, DNA, Genotype, Measurement error, Quality control, Whole genome amplification

## Abstract

**Background:**

Genotyping has become more cost-effective and less invasive with the use of buccal cell sampling. However, low or fragmented DNA yields from buccal cells collected using FTA cards often requires additional whole genome amplification to produce sufficient DNA for genotyping. In our case–control study of childhood leukaemia, discordance was found between genotypes derived from blood and whole genome amplified FTA buccal DNA samples. We aimed to develop a user-friendly method to correct for this genotype misclassification, as existing methods were not suitable for use in our study.

**Methods:**

Discordance between the results of blood and buccal-derived DNA was assessed in childhood leukaemia cases who had both blood and FTA buccal samples. A method based on applying misclassification probabilities to measured data and combining results using multiple imputations, was devised to correct for error in the genotypes of control subjects, for whom only buccal samples were available, to minimize bias in the odds ratios in the case–control analysis.

**Results:**

Application of the correction method to synthetic datasets showed it was effective in producing correct odds ratios from data with known misclassification. Moreover, when applied to each of six bi-allelic loci, correction altered the odds ratios in the logically anticipated manner given the degree and direction of the misclassification revealed by the investigations in cases. The precision of the effect estimates decreased with decreasing size of the misclassification data set.

**Conclusions:**

Bias arising from differential genotype misclassification can be reduced by correcting results using this method whenever data on concordance of genotyping results with those from a different and probably better DNA source are available.

## Background

The Australian Study of causes of Acute Lymphoblastic Leukaemia in Children (Aus-ALL) was a population-based case–control study, conducted between 2003 and 2007, designed to investigate environmental and genetic risk factors for childhood ALL as well as their interactions. The study has been described in detail elsewhere [1]. Briefly, 415 case children provided blood samples for genetic analysis during a routine visit to the treating hospital after initial remission was achieved. Buccal cell samples were collected from 536 control children at home using Whatman FTA Indicating Micro Cards (Cat. No.WB120211) (hereafter referred to as ‘FTA cards’) to maximize participation and minimize costs [2,3]. Whole genome amplification (WGA) from 1.2 mm diameter trephine punched discs were used to increase and preserve the finite amount of DNA available from the FTA card samples. Case children were also asked to provide a buccal sample using an FTA card so that concordance between genotypes measured using blood and FTA card buccal samples could be examined.

In this paper, we briefly present the concordance results for a set of six polymorphisms and describe the performance of a method we developed to correct genetic association analyses for the unexpected misclassification we observed. Because of the differential nature of the misclassification (all controls provided buccal DNA while most cases provided a blood sample) and the large number of covariates required and analyses planned, particularly all those requiring more than one gene in a model or examination of gene-environment or gene-gene interactions, we needed a comparatively simple and easy-to-use method for addressing this issue. While there is a considerable literature on methods for correction of measurement error or misclassification (for example, Guolo 2008 [4], and Thurigen et al., 2000 [5]), there appeared to be none that met our requirements with easily useable software. For example, the seismic program from Fox et al. [6] only handled one binary misclassified variable, and the Mime approach of Cole et al. [7] handled only one binary misclassified variable and required some of the validation sample to also include controls. The more flexible MC-SIMEX method of Kuchenhoff et al. [8], available in the R-library, was also not considered to be suitable, as the corrected results took no account of the size of the validation sample and was highly dependent on the choice of the extrapolation function. Clayton et al. have described an ingenious method for adjusting for a similar problem of differential bias [9], which uses statistical properties of the observed data and requires no validation data; however, it requires large samples so could not be used in our study. Thus it was necessary to develop a novel correction method, as described below.

## Methods

### Laboratory methods

The blood samples from case children were couriered to the processing laboratory overnight following collection. Whole blood was refrigerated at 4°C for a maximum of 7 days prior to DNA extraction using the Wizard Genomic DNA Purification Kit (Promega, Madison WI cat #1620) in accordance with the manufacturer’s instructions. DNA concentrations were quantified using a ND-1000 spectrophotometer (Nanodrop Technologies, Wilmington DE). FTA cards were stored at room temperature with desiccant until processing. Four discs of 1.2 mm diameter were trephine punched from the sampling area and placed collectively into a single tube and amplified using the GenomiPhi DNA Amplification Kit (GE Healthcare, Buckinghamshire UK). Briefly, 9μL of sample buffer, 9μL of reaction buffer and 1μL of enzyme mix were added to the punches with thermo cycling conditions according to the manufacturer’s instructions. DNA concentrations were quantified using a ND-3300 fluorospectrometer. The quantified DNA aliquots were then frozen at minus 30 degrees and thawed when required for genotyping.

Genotyping for single nucleotide polymorphisms (SNPs) involved in folate-metabolism, xenobiotic biotransformation and DNA repair pathways was performed using either restriction fragment length polymorphism analysis or TaqMan® SNP Genotyping Assays (Applied Bios stems, Foster City USA) with allele calling was performed by two independent researchers. For quality assurance purposes, 10% of samples were selected at random for repeat analysis, performed by laboratory staff blinded to sample identity. The specific SNPs genotyped in Aus-ALL are not identified in this methodological paper, as they are used for illustration purposes only. Full analyses of their associations with childhood ALL will be published elsewhere.

### Genotyping performance

#### All Aus-ALL SNPs

Across all 26 genotyped SNPs, the overall concordance within runs (repeat analyses of same sample) was 99.7% for blood samples (genomic DNA, hereafter referred to as gDNA) and 99.6% for WGA’d buccal DNA from the FTA cards (hereafter referred to as wgaDNA). The overall genotype failure rates for these SNPs were 0.16% and 1.68% for the gDNA and wgaDNA genotyping respectively. There were no departures from Hardy-Weinberg predictions observed in either the gDNA or wgaDNA genotype results. Among pairs of gDNA and wgaDNA samples collected from 249 case children, the average genotype discordance across all 26 diallelic SNPs (excluding discordance due to genotyping failure of one of the samples) was 3.48%, with a range of 0.40% to 12.96%. This discordance was the reason we developed the adjustment method described in this paper.

It appeared that the most likely source of error was preferential amplification from one chromosome of a pair, leading to loss of heterozygosis or “allelic drop-out” during WGA of frozen-thawed buccal DNA samples extracted from the FTA cards. Only WGA’d buccal DNA was available for control subjects; thus allelic drop-out in control subjects’ genotypes could bias associations between genotypes (and genotype-exposure interactions) and risk of disease. A modified unconditional logistic regression modeling technique was therefore developed to correct for this bias.

We assumed the genotype from the gDNA sample was the ‘true’ genotype (gold standard). 92.2% of the genotyping error in the wgaDNA samples involved a change from heterozygous in the gDNA to homozygous wild type (Aa→AA) or homozygous mutant in the wgaDNA (Aa→aa) (Table 1). Only 7.8% of discordant pairs involved a gDNA homozygous result (AA or aa) and a wgaDNA heterozygous result (Aa).

**Table 1:** Discordance in Genotyping Results Derived From gDNA and wgaDNA Samples From Case Children (excluding discordance due to failure of one sample)

**ID**	**N pairs**	**N Discordant**	**% Discordance**	**Aa to AA**	**Aa to aa**	**AA to Aa**	**AA to aa**	**aa to Aa**	**aa to AA**
A	213	5	2.35	1	3	1			
B	247	32	12.96	15	14	2		1	
C	246	12	4.88	4	7	1			
D	249	9	3.61	2	7				
E	243	18	7.41	7	10			1	
F	248	1	0.40	1					
All SNPs	1446	77	5.3%	30 (39.0%)	41 (53.2%)	4 (5.2%)	0	2 (2.6%)	0
				Total from Aa: 71 (92.2)		Total from AA: 4 (5.2)		Total from aa: 2 (2.6)	

N pairs: number of cases that had gDNA and wgaDNA sample to compare. N discordant: total number of discordant results. Aa to AA: discordance defined as heterozygous in gDNA sample but homozygous wildtype in wgaDNA sample. Aa to aa: discordance defined as heterozygous in gDNA sample but homozygous mutant in wgaDNA sample etc.

#### Algorithm testing subset

A subset of 6 SNPs from the Aus-ALL study was selected for use in the testing of the devised algorithm. These SNPs were selected as they covered the full spectrum of gDNA-wgaDNA genotype discordance seen. The mean level of discordance (excluding discordance due to genotyping failure of one of the samples) for these SNPs was 5.3% (range 0.40% to 12.96%).

### Statistical correction method

In brief, a discordance table is created by comparing the genotyping method that has error with a gold standard method. If discordance is present, then a proportion (calculated from the discordance table) of the records with genotypes containing error are randomly selected, their genotypes are corrected and a logistic regression model is computed. This process is repeated multiple times, ensuring a different set of records is randomly selected each time, and controlling for the fact that the proportion is an estimate. Results from the multiple iterations of the model are then compiled and imputed effect estimates are calculated. SNP A from Table 1 is referred to throughout the description of this algorithm as an example

#### Stage 1: Establish discordance tables

The proportions of discordant results for each SNP in the paired gDNA-wgaDNA samples are shown in Table 1. We calculated the proportions of homozygous wild type (AA), heterozygous (Aa) and homozygous mutant (aa) results from the wgaDNA samples that differed from the paired gDNA sample results, and used these to estimate the true misclassification proportion in Stage 2 (see below).

Table 2 shows the number of wgaDNA samples within each genotype category that were misclassified (assuming the paired gDNA sample gave the true result). For example, for SNP A there was 1 (of 96) incorrect AA result (should have been Aa), 1 (of 91) incorrect Aa result (should have been AA) and 3 (of 26) incorrect aa results (should have been Aa).

**Table 2:** Discordance Between Genotyping Results in gDNA-wgaDNA Pairs observed in SNP A and B

			**Blood result**			
			**AA**	**Aa**	**aa**	**Total**
SNP A		AA	95	1	0	96
	Buccal result	Aa	1	90	0	91
		aa	0	3	23	26
		Total	96	94	23	213
SNP B		AA	96	15	0	111
	Buccal result	Aa	2	87	1	90
		aa	0	14	32	46
		Total	98	116	33	247

*AA*: Homozygous wildtype, *Aa*: heterozygous; *aa*: homozygous mutant genotype.

#### Stage 2: Correct the genotypes for analysis

The discordance tables prepared in Stage 1 are only an estimate, from one sample, of the true misclassification proportion *p*. Therefore, the proportion of records to be corrected can be sampled from a binomial distribution with probability of success parameter *p*, which is automatically calculated from the discordance table. To make this correction within control subjects, the genotyping results were treated as follows:

1. The SNP genotype variable was re-assigned to a temporary new genotype variable.

2. A single value (${\hat{p}}_{i}$) was sampled from a binomial distribution with probability of success parameter *p* (e.g. 1/96 for AA to Aa).

3. Using this sampled proportion, the number of required changes (*c*_*T*_) was determined by multiplying this proportion (${\hat{p}}_{i}$) by the total number of controls with the genotype (AA).

4. From the control subjects with genotype (AA), the calculated number (*c*_*T*_) of subjects which required genotype reclassification were randomly sampled (equal likelihood of selection, without replacement).

5. In the temporary new genotype variable, those subjects’ genotypes were then reclassified (from AA to Aa).

6. Steps 2 through 5 were repeated for each instance of misclassification (e.g. each non diagonal cell of the discordance table with a value other than 0)

Where two changes were required within one genotype for a SNP (for example SNP B, where some Aa values require reclassification to AA while others require reclassification to aa), the following 6 steps replaced steps 2 through 6 in the above process.

2. A single value (${\hat{p}}_{i}$) was sampled from a binomial distribution with probability of success parameter *p* (e.g. (2 + 1)/90 [2/90 for Aa to AA + 1/90 for Aa to aa]).

3. Using this sampled proportion, the total number of required changes (*c*_*T*_) was determined by multiplying this proportion (${\hat{p}}_{i}$) by the total number of controls with genotype (Aa)

4. From the control subjects with genotype (Aa), the number (*c*_*T*_) of subjects requiring genotype reclassification were randomly sampled (equal likelihood of selection, without replacement).

5. Then *p*_1_ and *p*_2_ were temporarily assigned as the proportions of *c*_*T*_ that were required for each change. Here *p*_1_ = 2/(2 + 1) for the reclassification of Aa to AA and *p*_2_ = 1/(2 + 1) for the reclassification of Aa to aa.

6. Temporary scalars *c*_1_ and *c*_2_ were then calculated (*c*_1_ = *p*_1_*c_T_ and *c*_2_ = *p*_2_*c_T_)

7. In the new genotype variable, *c*_1_ and *c*_2_ of the randomly selected subjects’ genotypes from step 4 were reclassified (*c*_1_ from Aa to AA and *c*_2_ Aa to aa).

8. Steps 2 through 7 were repeated for each instance of this type of misclassification (e.g. where a single row or column of the misclassification table had more than one non diagonal cell with a value other than 0)

#### Stage 3: Run model to generate ‘corrected’ odds ratios for the association between each SNP and ALL risk

Correction of the misclassified genotypes, as outlined in Stage 2, was then done in each run of an unconditional logistic regression model that was repeated 50 times. Relevant covariates were included in the model. For each variable in the model, the mean ß coefficient from the 50 iterations was used as the best estimate of the true coefficient. These estimates were used to calculate Wald Test p-values [10] and to generate 95% confidence intervals (CI) using Rubin’s estimate of variance for multiple imputation [7]. Odds ratios were obtained by exponentiation of the estimates.

#### Implementation

The algorithm outlined in this paper was implemented in the statistical package R. For the R code to implement the outlined method, see our institutes website [11] or Additional file 1. The functions that make up the implementation of this algorithm are written so that they can be applied to any type of problem with a dichotomous outcome (e.g case/control analysis), a two or three level categorical predictor variable (e.g. a genotype) and discordance data (e.g. from a subset of the records with data from a gold standard measure, as here, or from other reference sources). The functions also allow for the inclusion of covariates in the model.

#### Testing the efficacy of the algorithm

To test the efficacy of the correction method, we created a series of hypothetical datasets that reflected our complete study dataset. These datasets were based on the genotype frequencies of two artificial SNPs: ‘SNP 1’ with an approximate dominant risk model (ORs fixed at: AA = 1.0, Aa = 1.01, aa = 2.04) and ‘SNP 2’ with an approximate log additive risk model (ORs fixed at: AA = 1.0, Aa = 1.30, aa = 1.68). The proportional amounts of misclassification shown in Aus-ALL SNPs A and B (the latter representing the SNP with the most discordance) among gDNA-wgaDNA pairs (Table 2) were then applied to artificial SNPs 1 and 2, resulting in six separate datasets (SNPs 1 and 2 with no error, with SNP A-type error, and with SNP B-type error). Odds ratios estimated from these data sets are shown in Table 3. We also simulated the effect of increasing the number of sample pairs available for assessing gDNA-wgaDNA misclassification. In this simulation, we used the SNP B-type misclassification figures seen in Table 2 and multiplied the observed numbers, in turn, by 0.5 and 5.

**Table 3:** Results of Correction Procedure on Hypothetical Datasets for Two Types of Misclassification (observed in SNPs A and B)

**Misclassification applied to data**			**‘Recessive’ artificial SNP 1**		**‘Log additive’ artificial SNP 2**	
			**OR**	**95% CI**^**a**^	**OR**	**95% CI**^**a**^
None^b^	Uncorrected	Aa	1.01	0.73, 1.41	1.30	0.94, 1.80
		aa	2.04	1.29, 3.21	1.67	1.03, 2.71
None	Corrected	Aa	1.01	0.73, 1.41	1.30	0.94, 1.80
		aa	2.04	1.29, 3.21	1.67	1.03, 2.71
SNP A-type	Uncorrected	Aa	1.07	0.77, 1.49	1.37	0.99, 1.91
		aa	1.82	1.17, 2.84	1.50	0.93, 2.40
	Corrected	Aa	1.03	0.73, 1.45	1.32	0.95, 1.85
		aa	2.11	1.29, 3.45	1.74	1.04, 2.91
SNP B-type	Uncorrected	Aa	1.49	1.06, 2.09	1.92	1.37, 2.68
		aa	1.66	1.08, 2.54	1.36	0.86, 2.15
	Corrected	Aa	1.01	0.70, 1.45	1.30	0.91, 1.85
		aa	1.99	1.21, 3.26	1.63	0.97, 2.75

*OR* = Odds ratio; *CI* = confidence interval; *Aa* = Heterozygous genotype; *aa* = Homozygous mutant genotype.

^a^ Confidence intervals based on Wald method for uncorrected estimates and percentile-based for corrected estimates.

^b^Free of genotyping error.

Using our case–control data, we then estimated odds ratios, with and without correction, for the associations between each SNP in Table 1 and risk of ALL adjusting for study matching variables (child’s age, sex, and state of residence) and ethnicity.

## Results

As expected, the results of the uncorrected (‘true’) and corrected analyses were identical (Table 3) when no error was present in the hypothetical data for artificial SNPs 1 and 2. When error was present, the estimated ORs deviated from the ‘true’ result, and did so to a greater extent when SNP B-type error was applied. For both artificial SNPs 1 and 2, the correction procedure adjusted the estimates in the direction of – and close to – the ‘true’ result. For example, the ‘true’ OR for the SNP 1 aa genotype was 2.04 (95% CI: 1.29, 3.21). With SNP A-type error introduced into the data, the uncorrected OR was 1.82 (95% CI: 1.17, 2.84) while the corrected OR was 2.11 (95% CI: 1.29, 3.45), close to the ‘true’ result. When SNP B-type error was introduced, the uncorrected odds ratio was even further from the ‘true’ estimate – 1.49 (95% CI: 1.06, 2.09); however, the corrected odds ratio was 1.99 (95% CI: 1.21, 3.26), close to the ‘true’ result.

As seen in Table 4, the odds ratios remained very similar, but the confidence intervals were somewhat wider or narrower when we simulated the effect of decreasing or increasing (respectively) the number of sample pairs available for assessing gDNA-wgaDNA misclassification.

**Table 4:** Results of Correction Procedure for Hypothetical Data on SNP B-type Error after Increasing Size of Misclassification Sample

	**‘Recessive’ artificial SNP 1**				**‘Log additive’ artificial SNP 2**			
	** Aa**		** aa**		** Aa**		** aa**	
Size increase of misclassification sample	OR	95% CI^a^	OR	95% CI^a^	OR	95% CI^a^	OR	95% CI^a^
0.5 x size	1.01	0.66, 1.55	1.98	1.15, 3.38	1.30	0.85, 1.99	1.63	0.93, 2.84
1 x	1.01	0.70, 1.45	1.99	1.21, 3.26	1.30	0.91, 1.85	1.63	0.97, 2.75
5x size	1.00	0.71, 1.41	2.00	1.26, 3.19	1.29	0.92, 1.80	1.65	1.01, 2.69

*OR* = Odds ratio; *CI* = confidence interval; *Aa* = Heterozygous genotype; *aa* = Homozygous mutant genotype.

^a^Confidence intervals based on Wald method for uncorrected estimates and percentile-based for corrected estimates.

### Iterations

To verify the optimal number of iterations provided, we used the highest level of discordance (SNP-B type error) on the artificial SNP 1, and completed 1000 repeats of 25, 50 and 100 iterations to examine the distribution of the resulting imputed OR for the aa genotype. 95% confidence intervals for the spread of this imputed OR were (1.96 – 2.10), (2.01 – 2.11) and (2.02 – 2.09) respectively. We felt the precision gained in 50 iterations compared to 25 was of value, and that the extra precision gained in 100 iterations compared to 50 was insufficient to warrant the observed modest increase in computation time. It should be noted that increasing the number of iterations would further increase the precision of the estimate, with the only expense being in added computation time.

### Correction of case–control study data

The uncorrected and corrected odds ratios and 95% confidence intervals for risk of ALL associated with each genotype are shown in Table 5.

**Table 5:** Results of Analysis of Genotype Associated with Risk of ALL: Uncorrected and Corrected Case–control Analyses

**ID**	**Geno-type**	**n cases/controls (uncorrected)**	**Uncorrected OR**	**95% CI**	**Mean n controls (corrected)**^**a**^	**Corrected OR**	**95% CI**
		**Max 276/420**					
A	AA	116/186	1.0	referent	186	1.0	referent
	Aa	129/184	1.16	0.83, 1.61	189	1.13	0.81, 1.57
	aa	30/41	1.19	0.70, 2.03	36	1.35	0.76, 2.39
B	AA	122/170	1.0	referent	151	1.0	referent
	Aa	119/154	1.13	0.80, 1.58	186	0.81	0.56, 1.56
	aa	31/52	0.82	0.49, 1.37	39	0.95	0.53, 1.70
C	AA	87/119	1.0	referent	116	1.0	referent
	Aa	122/193	0.85	0.59, 1.23	208	0.76	0.52, 1.10
	aa	64/82	1.05	0.68, 1.63	71	1.16	0.73, 1.83
D	AA	174/262	1.0	referent	259	1.0	referent
	Aa	95/126	1.15	0.83, 1.61	136	1.04	0.74, 1.45
	aa	6/13	0.73	0.27, 1.97	6	1.74	0.44, 6.94
E	AA	62/78	1.0	referent	70	1.0	referent
	Aa	146/176	1.11	0.73, 1.68	205	0.84	0.54, 1.29
	aa	62/143	0.58	0.37, 0.92	122	0.59	0.37, 0.97
F	AA	204/315	1.0	referent	314	1.0	referent
	Aa	68/82	1.30	0.89, 1.89	83	1.26	0.87, 1.84
	aa	2/13	0.23	0.05, 1.06	13	0.23	0.05, 1.05

*CI*: Confidence interval; *OR*: odds ratio.

^a^ Mean number of controls with each genotype, after correction, based on one simulation of 50 iterations.

Almost 40% of the discordance in the gDNA-wgaDNA sample pairs was in the Aa→AA direction (Table 1). The effect of our correction was to reduce the odds ratio for the Aa genotype in all six SNPs (Table 5). Over 50% of the discordance seen in the gDNA-wgaDNA sample pairs was in the Aa→aa direction (Table 1). Correction increased the ORs for aa genotypes in four out of six SNPs, while the other two remained the same (Table 5).

## Discussion

We aimed to correct for bias introduced by differential error in the genotyping results for control subjects compared with case subjects in our case–control study of childhood ALL. Over 90% of discordance observed in the case gDNA-wgaDNA sample pairs involved a loss of the Aa genotype in the wgaDNA samples (Table 1), consistent with allelic drop out during the WGA process. Croft and colleagues reported loss of the heterozygote genotype in 60 to 91% of discordant gDNA and wgaDNA sample pairs [12]. If the wgaDNA samples of control subjects were affected in a similar way to, and to a similar extent as, those of the cases – as is probable since DNA from case children was taken at remission – the most likely effect of the presumed allelic dropout would be to produce a spuriously lower proportion of Aa calls among controls than among cases for particular SNPs. The effect of this would be to artificially inflate the OR associated with the Aa genotype relative to the AA.

Our method for correcting logistic regression results for genotyping error can be applied to any study where misclassification is known to be present in genotypes (or other types two or three level categorical data), and where validation data are available, or where informed estimates of probable misclassification can be made. The method can also be adapted to allow associations between gene-environment interactions and risk of disease to be investigated.

Our correction technique involved using the empirically observed patterns of discordance in matched gDNA and wgaDNA samples from cases to adjust the estimated ORs for the associations between each of these SNPs and the risk of ALL. As expected, the primary effect of the correction on real data was to reduce the ORs associated with the Aa genotype. It also tended, less predictably, to increase the ORs for aa genotypes. There are two likely explanations for this less predictable effect. First, the aa genotype is generally the rarest so that even small adjustments in frequency can produce relatively large proportional changes in the OR. Second, for most SNPs, the correction method involved simultaneous adjustment to the frequency of the AA genotype relative to the Aa genotype, so the reference category for the aa OR was also adjusted.

Applying the discordance seen in our quality control sample pairs to two artificial SNPs showed that the method was successful: when the correction was applied to hypothetical data with a known degree of misclassification, estimates were more similar to those of the error-free hypothetical data. This was the case whether the degree of misclassification was relatively small (SNP A-type) or relatively large (SNP B-type). Although the effect of the correction method on the ORs for the aa genotype tended to be less predictable than for the Aa genotype when the ‘true’ result was unknown, our simulations showed that the corrections made to the aa genotypes were as appropriate and effective as for the Aa genotypes. These results give us confidence that the correction method we devised can adjust appropriately for the genotyping error and, therefore, should reduce bias in our estimated ORs. However, since the adjustment is probabilistic, there is likely to be a reduction in precision. Simulating an increase or decrease in the size of the misclassification data set narrowed or widened (respectively) the confidence intervals (Table 4).

## Conclusions

The statistical method we have described in this paper provides a novel and user-friendly method of correcting for differential genotyping error. Unlike previously described approaches, it can be used with modest sample sizes, allows correction of multiple misclassified variables, takes account of the size of the validation sample, and does not require the validation sample to include controls. Other genetic association studies based on cases and controls with samples derived from different sources or treatments of DNA should seek to investigate concordance between genotypes measured in DNA from both sources and, if found to be materially discordant, consider applying this adjustment procedure.

## Abbreviations

ALL: Acute lymphoblastic leukaemia; DNA: Deoxyribonucleic acid; OR: Odds ratio; SNP: Single nucleotide polymorphism; WGA: Whole genome amplification.

## Competing interests

The authors declare that they have no competing interests.

## Authors’ contributions

MC devised and coded the statistical method, and drafted the paper, NDK advised on statistical methods, and drafted the paper, EM was involved in study design and management and drafting the paper, KG ran the analysis of real and mock genotyping data and assisted in drafting the paper, SJ advised on genotyping analysis and methods, DA advised on statistical methods, FB coordinated sample processing, genotyping methods, study design and assisted in drafting the paper, BA was involved in study design and drafting the paper. All authors approved the final version of the paper.

## Pre-publication history

The pre-publication history for this paper can be accessed here:

http://www.biomedcentral.com/1471-2288/12/141/prepub

## Supplementary Material

Additional file 1 **R functions for misclassification correction method.** A text file containing the R functions required to run the logistic regression corrected for known misclassification, as outlined within this manuscript. Click here for file
